# Translation of two healthy eating and active living support programs for parents of 2–6 year old children: a parallel partially randomised preference trial protocol (the ‘time for healthy habits’ trial)

**DOI:** 10.1186/s12889-020-08526-7

**Published:** 2020-05-07

**Authors:** Megan L. Hammersley, Rebecca J. Wyse, Rachel A. Jones, Luke Wolfenden, Serene Yoong, Fiona Stacey, Simon Eckermann, Anthony D. Okely, Christine Innes-Hughes, Vincy Li, Amanda Green, Christine May, Joe Xu, Chris Rissel

**Affiliations:** 1 Early Start, Faculty of Social Sciences, University of Wollongong, Northfields Ave, Wollongong, NSW 2522 Australia; 2 Illawarra Health and Medical Research Institute, Northfields Ave, Wollongong, NSW 2522 Australia; 3 School of Medicine and Public Health, University of Newcastle, University Drive, Callaghan, NSW 2308 Australia; 4 Hunter New England Population Health, Locked Bag 10, Wallsend, NSW 2287 Australia; 5 Hunter Medical Research Institute, 1/Kookaburra Circuit, New Lambton Heights, NSW 2305 Australia; 6 Priority Research Centre for Heath Behaviour, University of Newcastle, University Drive, Callaghan, NSW 2308 Australia; 7 Australian Health Services Research Institute, University of Wollongong, Northfields Ave, Wollongong, NSW 2522 Australia; 8 NSW Office of Preventive Health, Liverpool Hospital, Locked Bag 7103, Liverpool BC, Sydney, NSW Australia; 9 Formerly Murrumbidgee Local Health District, Cootamundra Health Service, McKay St, Cootamundra, NSW Australia; 10 Sydney School of Public Health, University of Sydney, Sydney, NSW 2006 Australia

**Keywords:** Childhood obesity prevention, Fruit, Vegetable, Intervention, Home food environment, Healthy eating, Screen time, Sedentary behaviour, Physical activity, Movement

## Abstract

**Background:**

Parents are key decision makers and role models in establishing and maintaining healthy behaviours in their children. Interventions involving parents have been shown to be more effective than those that do not, but there are barriers to participation. Efficacy trials have previously been conducted on two such parent-focussed healthy eating and active living interventions with the potential to overcome these barriers - *Healthy Habits* (telephone-based) and *Time2bHealthy* (online) with promising results. Further research is now required to determine the effectiveness of these interventions in a real-world context. The *Time for Healthy Habits* study is a 3-arm partially randomised preference trial which aims to evaluate the effectiveness and cost-effectiveness of two theory-based programs to promote healthy eating and appropriate levels of movement behaviours (physical activity, sedentary behaviour and sleep) for parents of 2- to 6-year-old children (*Healthy Habits Plus* telephone-based program and *Time2bHealthy* online program), when compared to a comparison group receiving written materials.

**Methods:**

Participants will be recruited across five Local Health Districts in New South Wales, Australia. The partially randomised preference design initially allows for participants to decide if they wish to be randomised or opt to select their preferred intervention and has been recommended for use to test effectiveness in a real-world setting. Both interventions incorporate multiple behaviour change techniques and support parents to improve their children’s healthy eating, and movement behaviours (physical activity, sedentary behaviour and sleep) and run for 12 weeks, followed by a 3-month and 9-month post-baseline follow-up. Participants will also be asked to complete a process evaluation questionnaire at the completion of the intervention (3-months post-baseline). Outcomes include fruit and vegetable intake (primary outcome), non-core food intake, weight status, physical activity, sedentary behaviour, and sleep habits.

**Discussion:**

To our knowledge, this is the first translational research trial evaluating the effectiveness and cost-effectiveness of a healthy eating and active living intervention in the 2- to 6-years age group. The results will build the evidence base in regard to translation of effective childhood obesity prevention interventions and inform the implementation and delivery of community based childhood obesity prevention programs.

**Trial registration:**

UTN: U1111–1228-9748, ACTRN: 12619000396123p.

## Background

Worldwide, overweight and obesity affects around 23% of children and adolescents in developed countries and 13% in developing countries [1]. The percentage of overweight and obese children and adolescents in Australia is just over one quarter [2]. Appropriate dietary intake, and appropriate levels of movement behaviours (physical activity, sedentary behaviour and sleep) are key factors in early childhood obesity prevention and in establishing habits and routines that are protective against obesity in adulthood [3].

In New South Wales, Australia, a coordinated approach to childhood obesity prevention has focussed on primary and secondary prevention programs delivered at scale in children’s settings as well as community based treatment [4]. Interventions targeting parents as key decision makers and role models in establishing and maintaining healthy behaviours in their children have the potential to complement this approach. Previous studies have demonstrated that childhood obesity interventions targeting parents have been more successful than those which have targeted only children [5–7], and involvement of parents in interventions targeting younger children appears critical [6, 8]. However, there are challenges which make it difficult for parents to become involved in face-to-face programs, such as travel [9], cost [5], childcare for other siblings [10] and finding the time to attend [5]. Online and telephone-based interventions have the potential to overcome some of these barriers, offering potential advantages of convenience and flexibility for parents, and enabling parents to participate regardless of their geographic location.

Efficacy trials have been conducted on two such interventions in preschool aged children: *Healthy Habits* (3–5 years) [11] and *Time2bHealthy* (2–5 years) [12]. *Healthy Habits* is a telephone-based intervention which consisted of 20–30 min telephone counselling calls delivered weekly over 4 weeks. The program was developed by a team of health promotion practitioners, dietitians, psychologists and parenting experts, and is based on the family-based model of intervention proposed by Golan, which draws on socio-ecological theory [13]. The intervention primarily focused on increasing fruit and vegetable consumption. Results from the efficacy trial demonstrated a significant improvement in child intake of fruits and vegetables at 6-month (*p* = 0.021) [14] and 12-month (*p* < 0.01) [15] follow-up. *Time2bHealthy* is an online intervention consisting of six modules delivered over a 3-month period. The intervention was developed by a multi-disciplinary team of health behaviour and parenting experts and practitioners and is guided by Bandura’s Social Cognitive Theory [16]. The efficacy trial found positive outcomes for several nutrition outcomes at the 6-month follow-up, namely a significant reduction in child discretionary food intake (*p* < 0.01), and improvements in nutrition parent self-efficacy (*p* = 0.01) and pressure to eat child feeding practices (*p* = 0.048) [17]. Both *Time2bHealthy* and *Healthy Habits* demonstrated very high levels of user acceptance [17, 18].

Due to promising results from both efficacy trials, this current research aims to build on these previous studies, and examine the effectiveness of the interventions when they are scaled up and adapted for implementation across five Local Health Districts (health jurisdictions) in New South Wales, Australia. Translational research is important to assess population uptake, and the extent to which the effects of the intervention can be replicated when delivered at scale and with more diverse population groups with different sample characteristics and more flexible delivery approaches [19–21]. It is important that the effect of interventions is assessed when delivered in a manner which mimics how they are envisaged to be provided in the community. As such translational intervention methods are likely to be more pragmatic and may be adapted to allow more flexible delivery and less stringent eligibility criteria, instead concentrating on establishing external validity [22]. Fidelity and dosage of an intervention in translational trials may also be lower than efficacy trials as participants may be less likely to adhere to the intervention procedures or there may be less training provided for real-world staff implementing an intervention [23]. Unsurprisingly, when obesity prevention interventions are scaled-up, there is generally a reduction in the intervention’s effect size on outcomes such as weight status, dietary intake, physical activity and sedentary behaviour, possibly due to lower fidelity to original intervention protocols and increased flexibility in delivery [24]. To date, few childhood interventions to promote healthy eating and appropriate levels of movement behaviours (physical activity, sedentary behaviour and sleep) have been translated and evaluated in real-world settings [25] and most of these translational studies have been conducted in school aged-children [26–28] or infants [29]. Studies using a preference trial design (where at least a proportion of the participants have the option of selecting their preferred study arm), have been conducted successfully in different population groups [30–32], but there have been no such studies investigating the effectiveness of a childhood obesity prevention intervention, or any focussing on obesity or obesity-related behaviours in any age group.

The aim of this research study is to examine the relative effectiveness and cost effectiveness of the *Time2bHealthy* and *Healthy Habits Plus* programs when they are applied to existing health services across metropolitan, regional, and rural New South Wales and offered as a free population-wide service across these jurisdictions to mimic real-world implementation.

The primary objectives of the study are to:

1. Estimate the relative effectiveness of an online behaviour change program (*Time2bHealthy*), a telephone-based support program (*Healthy Habits Plus*) or a written material active control in targeting parents of 2–6 year olds in changing child fruit and vegetable consumption (primary outcome).
2. Estimate the relative effectiveness, cost and cost effectiveness of Time2bHealthy, Healthy Habits Plus and the active control with respect to non-core food consumption, levels of movement behaviours (physical activity, sedentary behaviour (including screen-time), sleep), and weight status effects in accordance with Australian guidelines (secondary outcomes).

The secondary objectives are to:

1. Explore the most successful approaches to maximise recruitment to these interventions, and the retention of parents within them.
2. Determine the preferred ex-ante and ex-post user delivery medium.

Robust evaluation of the relative incremental effects, costs and cost effectiveness of health promotion strategies in community settings such as Healthy Habits (telephone-based) and Time2bHealthy (online) needs to consider:

(i) at an individual level the a priori relative preferences for and uptake of strategies in target community populations of parents [33–36];
(ii) the effects on children and parents exposed to strategies and;
(iii) at a community level the community ownership and potential for network and multiplier effects that health promotion programs uniquely enable [37–40].

This study aims to collect trial evidence at an individual level in addressing the first two questions and to facilitate triangulation of evidence in modelling potential of the third.

## Methods

### Study setting

The study is being conducted in New South Wales, Australia and the Local Health Districts of Murrumbidgee, Hunter New England, Illawarra Shoalhaven, Southern New South Wales and South Eastern Sydney are being specifically targeted for recruitment. Together, over 2.6 million people live in these Local Health Districts and they provide a representative sample of young children, with over 300,000 children in the 0–9 years age bracket [41].

### Eligibility criteria

Parents will be eligible if they live in New South Wales, have a child 2–6 years of age at the time of the baseline interview, live with the child at least 4 days on an average week, have access to telephone and internet, and understand and speak English sufficiently to participate. Parents will be excluded if they have previously participated in the *Time2bHealthy* or *Healthy Habits* randomised controlled trials (RCTs).

### Study design

This protocol was written in accordance with the SPIRIT (Standard Protocol Items: Recommendations for Intervention Trials) statement [42]. A three-arm parallel-group randomised preference trial design will be used. The three arms comprise; (1) the *Healthy Habits Plus* telephone-based intervention, (2) the *Time2bHealthy* online intervention, and (3) a comparison arm, which will consist of printed educational material.

To address the question of individuals’ a priori preferences in the target population, as well as the relative effects in children and families, the trial recruitment will initially offer parent participants the option of receiving their preferred delivery medium or be randomised. The preference trial design is particularly well suited to examining the effectiveness of interventions conducted in real-world contexts as it can mimic usual service provision circumstances and allow an estimate of the intervention effects of those who would typically utilise a specific intervention if it was made available. While RCTs are the recognised gold standard for efficacy trials, when the participant has a strong preference in regard to the arm of the study they would choose, they may either be reluctant to be involved if they know they will be randomised, or if they do opt to be involved, they may drop out or not carry out the required activities of the intervention if they are not assigned to their preferred study arm [33–36, 43, 44]. The partially randomised preference trial design allows parents who have a strong preference for the intervention they receive to elect the group to which they are allocated. Parents who do not have a strong preference will be randomly allocated to a group in a 1:1:1 ratio. This study design has been used in previous implementation and translational research [30, 32]. Importantly it can answer the research question regarding which intervention is preferred by parents through the percentage of participants opting to choose each of the interventions.

The study design has a higher likelihood of initial acceptability by participants, if they know that they will be able to choose their preferred study arm, optimising recruitment efforts. Preference allocation can also better reflect real life community preferences and uptake across health promotion strategy alternatives compared with randomising individuals. In a traditional RCT design attempts to randomised individuals can pose recruitment challenges, and population biases with dropout can arise in community settings where participants have a preference for or against a study arm [33–36, 43, 44]. These issues have potential to compromise the generalisability and internal and external validity of individual randomised RCT studies [33, 35, 36]. Despite potential recruitment and retention advantages of preference design over RCTs with randomised individuals, relative effects between arms can be confounded with preference design to the extent there are selection bias between participants who opt to choose alternative intervention arms. To avoid potential for such biases the primary analysis of individual effects for this study will be conducted on participants in the randomised group only (with uptake and secondary analyses being conducted on the entire sample including preference group participants). Stopping rules for the non-randomised preference arm will be applied to ensure enough participants are randomised to power the primary analysis of relative treatment effects.

### Interventions

#### Healthy habits plus

*Healthy Habits Plus* is a telephone-based intervention, which consists of six 20–30 min telephone counselling calls delivered fortnightly over 3 months by trained para-professionals (who have experience in conducting health-related telephone interviews and surveys, but do not necessarily have formal qualifications in a health profession).

The program has been updated for this trial from its original 4-call format focussing exclusively on healthy eating (*Healthy Habits*), to reflect recent guideline updates, and also to incorporate content on movement behaviours (physical activity, screen time and sleep). The program seeks to improve healthy eating, and levels of movement behaviours (physical activity, sedentary screen time and sleep) through modifying: i) the availability and accessibility of foods and beverages, (i.e. ensuring fruit and vegetables are present and stored in a ready-to-eat form), opportunities for physical activity in the home and limiting the presence of screens/devices; ii) supportive family routines (i.e. eating meals without the television, having a set bedtime) and iii) parental role-modelling of health behaviours. Such factors are associated with obesity-related behaviours in young children [45–50].

Consistent with the original intervention and to support behaviour change, the telephone counselling intervention utilises a number of specific behaviour change techniques including barrier identification, goal-setting, self-monitoring, and using prompts or cues [51]. Parents will be mailed print materials (i.e. a guidebook and pad of menu planners) to be used during the telephone contacts and to facilitate action between calls.

To maximise adherence to the intervention, up to 10 attempted calls will be made to participants at each scheduled call, leaving a voice message at the first opportunity. These calls may be complemented by SMS and email as additional measures to contact the participant. After multiple unsuccessful contact attempts, an SMS will sent to ask participants if they would like to continue to participate (Y or N). If they respond ‘N’, they will be withdrawn from the study.

#### Time2bHealthy

*Time2bHealthy* is an online intervention that consists of six modules, each taking approximately 30 min for parents to complete and are delivered over 3 months (i.e. one module every 2 weeks). The program seeks to improve child healthy eating as well as movement behaviours (physical activity, sedentary screen time and sleep), through targeting characteristics of the home environment, developing supportive routines, and encouraging parental modelling of health behaviours. Parents will be required to read content, complete practical activities, watch videos and set goals within each module to facilitate behaviour change. The intervention will incorporate a closed Facebook group (moderated by a health professional) which will allow participants the opportunity to communicate with other members of the intervention cohort (consisting of approximately 15–25 participants per cohort).

To maximise adherence to the intervention, participants will receive an email each week reminding them to log onto the website to complete the modules. One to two moderator posts will be placed in the Facebook group each week reminding participants to log onto the websites and to contribute to the Facebook discussion.

#### Comparison group

The comparison group will receive written educational materials on standard recommendations for healthy eating, and movement behaviours (physical activity, sedentary screen time and sleep). The educational materials, developed by the NSW Office of Preventive Health, also encourage parents to access the *NSW Healthy Kids* and *Raising Children Network* websites. These materials will be supplied to parents at fortnightly intervals over a 3-month period via email or post (based on preference).

### Outcomes

#### Primary outcome

##### Child fruit and vegetable intake patterns

The primary trial outcome is child fruit and vegetable intake patterns, as determined through their score on the fruit and vegetable subscale of the Children’s Dietary Questionnaire (CDQ). The subscale is scored from 0 to 28 with a score of 14 or more recommended based on the Dietary Guidelines for Children and Adolescents [52] and the Australian Guide to Healthy Eating [53].

The questionnaire assesses fruit and vegetable intake patterns over both the past week and 24 h. Acceptable reliability and validity has been established for this questionnaire in assessing child dietary patterns among preschool children at a population level, and for use in assessing the efficacy to improve children’s eating habits [54].

As the CDQ does not assess number of servings, crude servings-based measures of child fruit and vegetable intake will also be used. These questions were taken from the New South Wales Child Health Survey and ask parents *“How many serves of fruit does [child name] usually eat each day?”* and *“How many serves of vegetables does [child name] usually eat each day?”* [55].

This outcome will be assessed by three means: firstly, the absolute change in fruit and vegetable intake will be assessed [55], secondly, the change in scores on the fruit and vegetable subscale of the CDQ [54] will be assessed, and finally, the change in compliance to fruit and vegetable guidelines will be assessed.

#### Secondary outcomes

##### Child non-core food intake

Absolute changes in child intake of non-core foods will be assessed based on the scores on the non-core foods subscale of the CDQ [54].

##### Child weight status

Child BMI percentile will be assessed based on parent-reported child height (m) and weight (kg) using standard items from the NSW Population Health Survey, which have been tested for reliability and convergent validity [56, 57].

##### Child physical activity and sedentary screen-time

Child physical activity will be assessed using a parent-reported questionnaire. Parents will be asked about the amount of time (in minutes) that their child was physically active during the whole day prior to the interview and specifically how much of this was of moderate or vigorous intensity. They will also be asked how many days over the past week their child was active for at least 3 hours. Parents will be asked about the amount of time (in minutes) that their child used electronic media devices (including TV, consoles and hand-held devices) while sitting or lying down during the whole day prior to the interview. They will also be asked how many days over the past week their child used such electronic media devices for less than 1 hour. These questions were modified from the National Nutrition and Physical Activity Survey (Australian Bureau of Statistics 2011) and are intended to assess compliance to the physical activity and screen-time components of the Australian 24-h Movement Guidelines for the Early Years. Compared with accelerometer measures, correlations between parent-reported measures of the percentage of time in sedentary, moderate, vigorous and moderate- to vigorous-intensity activity ranged from *r* = 0.35 to 0.49 [58] and it is therefore considered acceptable to use parent-reported data to assess physical activity and sedentary time.

##### Child sleep

The modified Children’s Sleep Habits Questionnaire will be used to assess child sleep duration and nap time and compliance with the sleep component of the Australian 24-h Movement Guidelines for the Early Years. The questionnaire items will also assess sleep reluctance. The questionnaire has been validated against accelerometry in samples that included preschool-aged children [59].

##### Mediators

Selected factors identified as key potential/hypothesised mediators of the interventions will be assessed, drawn from items in the Healthy Home Survey (HHS) [60]. These include the following: fruit and vegetable availability (i.e. available from the home environment), fruit and vegetable accessibility (i.e. able to be accessed in home environment - fruit and vegetables are within reach in ready to eat form), providing behaviour of parents, and family mealtime practices (family eating dinner at table together, not watching TV while eating a meal). These items were previously identified as potential mediators in the *Healthy Habits* efficacy trial [61].

##### Cost and cost effectiveness

Alongside relative study effects on primary (fruit and vegetable) and secondary outcomes (non-core food, physical activity, sleep, screen time and sedentary behaviours) information about resource use and costs will be collected from intervention and comparison arms. Resource use and costs of strategies across arms include staff time and capital costs originating at program, community and individual level. Staff time covers recruitment of participants, arrangement and delivery of the interventions (telephone interviewer and Facebook group facilitator time and program administration) costed at the relevant wage rates (including penalty rates and on-costs). Within trial relative costs of alternative strategies over 9-month follow-up will be estimated from observed resources used in implementation at a participant and local health district level applying relevant prices**.** Population level joint distribution of cost and effects under uncertainty will be jointly considered under uncertainty employing bootstrapping at individual and local health district level in net benefit assessment. This approach allows robust estimation of within study joint distribution under uncertainty of the relationship (covariance) between costs and effects observed in the randomised population [62].

Longer term effects and costs of strategies will be estimated triangulating across trial evidence of individual and community uptake, dose and effects from population exposure, indication of strategy continuation beyond trial, and wider community uptake of strategies. Triangulation of evidence is key to satisfy comparability and coverage in robustly informing societal decision makers of the relative net benefit of strategies under uncertainty in evaluating the effectiveness and cost effectiveness of health promotion interventions [63–65].

##### Participant preference

One of the objectives of the study is to determine the preferred user delivery medium. This will be assessed two ways. Ex-ante preferences will be determined at the conclusion of baseline data collection, where participants will be asked if they have a strong preference in regard to the way that they receive information and if so, what their preferred medium is (telephone, online or written materials). Ex-post preferences will be determined at the conclusion of the 3-month follow-up, where all participants will be asked whether they would have preferred for the information to be delivered by a different mechanism. They will be provided with the following options; telephone counselling, online program, educational materials, smartphone app, face-to-face, Skype, or other.

##### Comparison of recruitment & retention strategies

Several recruitment strategies will be employed by Local Health District staff at a local level and other broad-level recruitment strategies will also be applied (refer to recruitment section for details). Local Health Districts will decide which of these strategies to employ depending on their capacity and/or existing relationships with agencies and the community. To determine the most successful strategies, participants will be asked a multiple-choice question about how they found out about the program at the baseline data collection interview. These data will also be used to determine if there are recruitment strategies which are associated with a higher retention rate. A standard spreadsheet will also be used by each Local Health District to capture data on face-to-face recruitment initiatives, detailing the number of sites attended, number of parents approached, and number providing consent which will assist in determining which face-to-face sites are most successful for recruiting participants to the study.

##### Fidelity of interventions

The delivery of the interventions will be monitored and the data used to determine if the interventions were delivered as intended. Number of scheduled calls completed will be used to measure the fidelity of the *Healthy Habits Plus* intervention. Number of modules completed will be used to measure the fidelity of the *Time2bHealthy* online intervention. Number of emails/mailouts sent will be used to measure the fidelity of the comparison (written) intervention.

### Participant timeline

There are three scheduled assessment time-points; baseline, 3-month (immediately post-intervention) and 9-month post baseline follow-up (approximately 6-months post-intervention). Participants will receive a phone call at the baseline and 9-month time-points. Data will be collected by trained telephone interviewers using Computer Assisted Telephone Interviewing (CATI). The questionnaire comprises questions as described in the outcome measures. At 9-month follow-up, data collectors will be blinded to the study arm that the participants are assigned to. To maximise retention, up to 15 attempted calls will be made to participants at baseline and follow-up, leaving a voice message at the first opportunity. These calls will be complemented by an SMS after the 4th and 9th call attempt. The SMS after the 9th call attempt will ask participants if they would like to participate (Y or N). If they respond ‘N’, they will be withdrawn from the study.

**Fig. 1:**
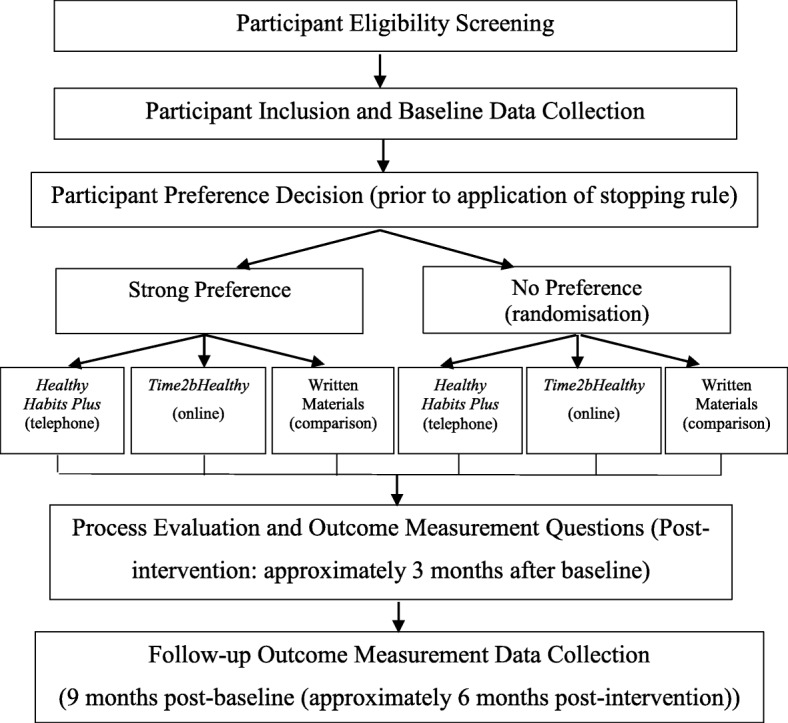
Study Flow Chart (prior to application of the stopping rule)

**Fig. 2:**
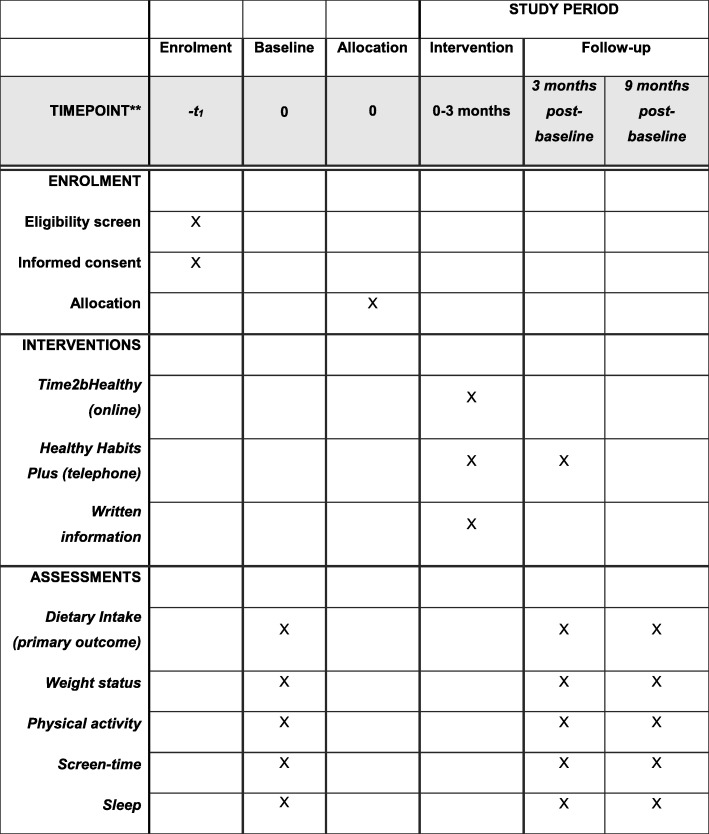
Time for Healthy Habits study schedule of enrolment, interventions, and assessments

The 3-month post-baseline assessment will include primary and secondary child behavioural outcome measure questions repeated from the baseline questionnaire, as well as a brief process evaluation. This will be completed either over the phone (for *Healthy Habits Plus* participants), online (for *Time2bHealthy* participants) or via mail/email (for comparison participants). The process evaluation will assess the user acceptance of the content and modality of each of the programs. The outcomes questions will include the fruit and vegetable subscale of the CDQ, and servings-based questions about fruit and vegetable intake (primary outcome). in addition to questions relating to the child’s physical activity, screen-time and sleep over the previous 24-h period (secondary outcomes). Figure 1 outlines the data collection time points and Fig. 2 provides an overview of the schedule of enrolment, interventions and assessments.

### Recruitment

A recruitment strategy was developed by the Project Steering Committee in consultation with the Participant Recruitment Working Group, consisting of key stakeholders from the NSW Office of Preventive Health, the University of Wollongong, the University of Newcastle and the Population Health division of each of the Local Health Districts participating in the study.

Each Local Health District has been actively involved in the recruitment planning process and in addition to the main aims of the study, the project also presents an opportunity to help to build research capacity in Local Health District health promotion staff. Each Local Health District will appoint a Recruitment Officer who will be responsible for recruitment in their local area, supported by a central Project Coordinator. The Recruitment Officers will conduct face-to-face visits to locations such as Early Childhood Education and Care Services, playgroups, clinics and early childhood activities to promote and discuss the study with parents and provide them with participant information sheets and consent forms, or give them the option to sign up to the study electronically. Recruitment officers will also discuss the study with health professionals in their area and ask them to refer any suitable parents to the study. Flyers and/or information sheets and consent forms will be provided to local health professionals to offer to patients. Promotional materials will also be provided to other key stakeholders in the local area and information will be communicated via local newsletters, community noticeboards, media releases, social media, and local networks. The study will also be promoted through a media release to local media outlets and to parenting groups on Facebook.

Parents will gain information and provide informed consent either through hard copy participant information sheets and consent forms provided by the Recruitment Officer or Project Coordinator or online through the study website https://timeforhealthyhabitsnsw.com

### Randomisation

Following provision of informed consent, participants will receive a phone call from a trained interviewer. A series of questions will be asked to confirm eligibility and baseline data will then be collected. Following baseline data collection, participants will be asked if they have a strong preference in regard to the way that they would like to receive information (via telephone, online or written). In the initial stages of the study, participants who have a strong preference will be given the option to choose their study arm, or otherwise be randomised in a 1:1:1 ratio. The study arm options will be presented in random order (to minimise the impact of participants choosing a group based on the order that the options are presented to them). Randomisation will be conducted using a random number function in SAS statistical software version 9.3 by an independent statistician who is not directly involved in the recruitment or analysis phases of the study. A stopping rule will be implemented, whereby participants will no longer be able to select their study arm if the prevalence of participants with strong treatment group preference precludes estimates of reasonable precision of intervention effects. Specifically, allocation will be ceased based on treatment preference and participants randomly assigned to each of the three experimental arms in the ratio (1:1:1) when, and if 285 (45%) of the anticipated 636 participants opt to select their study arm. This threshold will ensure that there are approximately 117 randomised participants per group.

### Data management

Participant contact details and details regarding management of participants through each of the interventions will be collected on the consent from and then entered and stored in REDcap data management software v 8.10.1 (Vanderbilt University) by the Project Coordinator and Data Manager. Participant data from baseline and follow-up data collection questionnaires will be entered in real-time by trained interviewers into the CATI system as the questionnaire takes place. Participant data will be stored on secured servers at the three project management sites - NSW Office of Preventive Health, the University of Wollongong (*Time2bHealthy* arm) and the University of Newcastle (*Healthy Habits Plus* arm), and backed up regularly. Data will be securely transferred between the sites where necessary.

Study data will be password-protected and will be accessible only to authorised members of the research team. Hard copies of signed consent forms will be stored in locked filing cabinets at the data collection sites. All participant data will be de-identified prior to any analysis and reporting.

Integrity of data will be managed by the use of valid values and ranges for parameters entered. To reduce risk of data entry error, CATI programming is in place which can prevent entry of values outside of the selected options for categorical data. For continuous data, reasonable value ranges are included in the programming that prompt interviewers to confirm that their response is correct before moving forward in the telephone interview. Due to the minimal risk involved in this study, a data monitoring committee was not established.

### Study design, sample size and study power

For the primary analysis, sample size calculations for the randomised population are based on detecting between group differences in the proportion of randomised children with fruit and vegetable intake patterns consistent with dietary guidelines, as determined by a score of 14 or more on the fruit and vegetable subscale of the CDQ [54], the primary outcome alone. Accounting for 20% attrition, 117 randomised participants per arm (351 in total) are needed to complete baseline data collection with adequate power, resulting in a sample size of 93 participants per group at the 9-month post- baseline follow-up. For 80% power at a 0.05 significance level, this will allow a 20% detectable difference between intervention and comparison groups in adherence to dietary guideline recommendations assessed using the CDQ fruit and vegetable subscale. The overall sample size of 636 participants was based on a more conservative effect size estimate of 15%.

### Statistical methods

#### Impact of intervention

All analyses will be conducted using intention-to-treat principles. Generalised estimating equations logistic regression models or mixed models will be used to assess the impact of the intervention on the primary trial outcome of the child fruit and vegetable intake. Secondary outcomes of non-core food intake, weight status, physical activity, sedentary screen time, and sleep will also be assessed. Intervention effects will be compared, firstly for participant level effect including only participants who were randomly allocated and secondly for a community level analysis including all participants adjusting for group (preference) and for other covariates. All significance tests will be 2-tailed with an alpha of 0.05. Post hoc analyses will be conducted to examine interactions by levels of socio-economic status. Exploratory analyses will also be conducted to determine whether preference modifies intervention effects.

#### Cost and cost effectiveness analysis

This trial is primarily designed to assess individuals’ preferences for and relative effects and costs of health promotion strategies within trial while providing some evidence towards community level evaluation in translating evidence to practice. Whole study modelled relative cost effectiveness evaluation at a community level given observed parent preferences and intervention costs of online, telephone-based and written interventions will be analysed synthesising pre-post effect change and cost data attributed to lowest level of analysis. Best multiple strategy and multiple domain comparison methods for societal decision making of pre-post change will be used to construct cost effectiveness acceptability curves and expected net loss curves and frontiers [38, 66, 67]. These summary measures best inform decision makers of the probability of being cost effective, differences in net benefit and the potential value of further research across plausible decision maker threshold values for effects across these strategies.

### Research ethics approval

This study was approved by the South Western Sydney Local Health District Human Research Ethics Committee (HE18/300) as well as site specific approval by Murrumbidgee Local Health District Human Research Ethics Committee, Hunter New England Local Health District Human Research Ethics Committee, Illawarra Shoalhaven Local Health District Human Research Ethics Committee, Southern New South Wales Local Health District Human Research Ethics Committee, and South Eastern Sydney Local Health District Human Research Ethics Committee, and acceptance by the University of Newcastle Human Research Ethics Committee (H-2019-0188) and the University of Wollongong Human Research Ethics Committee (HE2019/207). The study is registered with the Australian New Zealand Clinical Trials Registry (ACTRN12619000396123p), an acceptable registry of the International Committee of Medical Journal Editors (ICMJE).

As previously outlined, information on the study is available through the recruitment officers, flyers and promotional material, participant information sheets and the study website. Parents have the opportunity to ask questions to the Recruitment Officers, Project Coordinator or health professionals about the study prior to consenting.

To protect the privacy of participants, all data will be stored securely with limited access. All participant data will be de-identified prior to any analysis and reporting. Results from this study will only be presented and published in an anonymous and aggregated way so individual participants will be not identifiable.

### Protocol amendments

All protocol amendments will be documented in the ANZCTR (12619000396123p).

## Discussion

To the best of the authors’ knowledge, the *Time for Healthy Habits* study is the first partially randomised preference trial investigating the effectiveness of a childhood obesity prevention intervention. It is also the first translational research trial evaluating the effectiveness and cost-effectiveness of a childhood obesity prevention intervention in the 2–6 years age group as delivered via key health promotion partners.

This paper provides a detailed overview of the methods to be used to determine the effectiveness and cost-effectiveness of the *Time2bHealthy* online program and the *Healthy Habits Plus* telephone intervention compared to a comparison group in improving dietary intake, physical activity, sedentary screen-time and sleep habits, and weight status in children. The study also seeks to determine the most successful approaches to recruitment and retention, and build research capacity in Local Health District health promotion staff.

The trial will provide valuable evidence on preferences for, and the effectiveness and cost-effectiveness of remotely delivered healthy lifestyle interventions for parents of young children and will be used for evidence-based decision making to inform large scale implementation and future delivery of government-funded childhood obesity prevention programs in this age group.

The study has a number of strengths. The coverage of effects and their duration across major lifestyle factors for obesity prevention (physical activity, sedentary behaviour and sleep and their combinations alongside diet) and the novel partially randomised preference trial design of the study offers advantages in enabling synthesis of preference and RCT evidence. Adequate coverage of duration and scope of effects is particularly important for a pragmatic trial aimed at robustly informing societal decision and policy making of the population level effectiveness and cost effectiveness of alternative health promotion strategies in community settings (Eckermann and McCaffrey 2017; McCaffery and Eckermann 2017; Sheill et al. 1995, 2008, 2018). In health promotion settings preference based design also has potential for some advantages of internal and external validity over traditional RCTs, given potential participants may decline to enrol in RCTs if they have a strong preference for study arm and do not want to be randomised into their non-preferred arm. The absence of these potential participants in trials can affect the external validity of the study as the sample is unlikely to be representative of the population and therefore generalisability of the study can be affected [44]. Furthermore, internal validity of such trials can be affected by participants who do choose to be randomised but are allocated to their non-preferred arm. Such participants may be less likely to be motivated to actively participate and/or follow the intervention through to completion [44].

Other strengths include the blinding of telephone data collectors (outcome assessors), and the accessibility of the study to all residents of NSW, regardless of geographic location. As all data are collected via telephone or online and the interventions are delivered by telephone, online or email/mail and no face-to-face contact is required, usual barriers to traditionally delivered studies such as travel, scheduling of appointments and childcare for other siblings are not applicable. Accessibility of the study in rural and remote areas is particularly important given the higher prevalence of overweight and obesity and limited services in many areas [68].

There are, however, some potential limitations of the study. It may be challenging to recruit participants to randomised arms of the study. While some studies have not reported any such difficulty [31, 69], one study reported that only 3% of participants consented to being randomised [30], highlighting the importance of the planned stopping rule for the current study. Providing participants with the option to choose their study arm has the potential to result in unbalanced participant numbers across the study arms, which may have statistical implications and limit the ability of the trial to draw strong conclusions.

## Conclusion

This paper provides a detailed overview of the *Time for Healthy Habits* translational research trial which aims to determine and compare the effectiveness and cost-effectiveness of two remotely-delivered healthy eating and active living interventions for parents of 2- to 6-year-old children. The results will strengthen the evidence base in regard to translation of effective childhood obesity prevention interventions, inform the implementation and delivery of community based childhood obesity prevention programs, and assist in advancing the science of patient preference trial designs.

## Supplementary information

**Additional file 1.Table 1.** Trial registration data set.

## Data Availability

Not applicable.

## Abbreviations

ANZCTR: Australian New Zealand Clinical Trials Registry
CATI: Computer Assisted Telephone Interviewing
CDQ: Children’s Dietary Questionnaire
RCT: Randomised Controlled Trial
SMS: Short Messaging Service
SPIRIT: Standard Protocol Items: Recommendations for Intervention Trials
