# Supplementary material for: Translation of two healthy eating and active living support programs for parents of 2–6 year old children: a parallel partially randomised preference trial protocol (the ‘time for healthy habits’ trial)
Source: BMC Public Health. 2020 May 7;20:636. doi: 10.1186/s12889-020-08526-7 (PMC7204000; doi:10.1186/s12889-020-08526-7)
Supplement: Supplementary file 1 — Additional file 1.Table 1. Trial registration data set. [file 12889_2020_8526_MOESM1_ESM.docx]

**Supplementary Table 1: Trial registration data set**

| Primary registry and trial identifying number | UTN: U1111-1228-9748  Australian New Zealand Clinical Trials Registry (an acceptable registry of the International Committee of Medical Journal Editors (ICMJE))  ACTRN: 12619000396123p |
| --- | --- |
| Date of registration in primary registry | 12/03/2019 |
| Secondary identifying numbers | N/A |
| Source(s) of monetary or material support | New South Wales Health Translational Research Grants Scheme |
| Primary sponsor | Murrumbidgee Local Health District |
| Secondary sponsor(s) | University of Newcastle  University of Wollongong  NSW Office of Preventive Health |
| Contact for public queries | Dr Megan Hammersley |
| Contact for scientific queries | Prof Chris Rissel |
| Public title | Time for Healthy Habits: Evaluating Two Healthy Eating and Active Living Support Programs for Parents of 2-6 Year Old Children |
| Countries of recruitment | Australia |
| Health condition(s) or problem(s) studied | Childhood Obesity |
| Intervention(s) | *Healthy Habits* *Plus* telephone-based intervention  *Time2bHealthy* online intervention |
| Key inclusion and exclusion criteria | Inclusion criteria:   - Child 2-6 years of age - Lives in New South Wales, Australia - Child resides with parent participating in study at least 4 days per week - Parent speaks and understands English - Parent has access to phone and internet   Exclusion criteria:   - Previous participation in *Healthy Habits* or *Time2bHealthy* randomised controlled trials |
| Study type | Interventional  Allocation: Partially randomised preference trial design  Primary purpose: prevention |
| Date of first enrolment |  |
| Target sample size | 636 (351 randomised) |
| Recruitment status | Recruiting |
| Primary outcome(s) | Child dietary intake (fruit and vegetable) |
| Key secondary outcomes | Child dietary intake (non-core foods)  Child weight status  Child physical activity  Child sedentary screen time  Child sleep  Mediators |
